# Maternal diets matter for children's dietary quality: Seasonal dietary diversity and animal‐source foods consumption in rural Timor‐Leste

**DOI:** 10.1111/mcn.13071

**Published:** 2020-08-05

**Authors:** Gianna Bonis‐Profumo, Natasha Stacey, Julie Brimblecombe

**Affiliations:** ^1^ Research Institute for the Environment and Livelihoods Charles Darwin University Ellengowan Drive Darwin Northern Territory 0909 Australia; ^2^ Department of Nutrition, Dietetics and Food, Faculty of Medicine, Nursing and Health Sciences Monash University 264 Ferntree Gully Road Notting Hill Victoria 3168 Australia

**Keywords:** dietary diversity, animal source foods, agroecological zones, seasonality, Timor‐Leste

## Abstract

Improving the dietary quality of women and children is essential to reduce all forms of malnutrition. In this study, we assessed seasonal child and maternal dietary diversity and consumption of animal‐source foods (ASF), using 1,236 observations from combined data collected among 167 mother–child dyads in rural Timor‐Leste. We used generalized linear and logistic mixed‐effects models to examine the dietary differentials of mothers and children in two agricultural livelihood zones and across the seasons, as well as to identify household and agroecological characteristics associated with children's dietary quality in relation to their mothers'. We found dietary quality to be marginally better in coastal than in mid‐altitude zones. However, women's diets were strikingly poor, and their intake of ASF was lower than among children. Mothers exhibited preferential allocation patterns of specific ASF, dairy products and eggs, to children. The intake of ASF was predicted by seasonality. Flesh foods and red meat were much more likely to be consumed during the dry season, when cultural ceremonies are often performed. We found a positive and strongly significant association between children's dietary indicators—dietary diversity score, minimum dietary diversity and ASF consumption, and those of their mothers'. Maternal dietary quality and educational attainment, more so than agroecological characteristics, were explanatory factors of children's diet. Our study highlights that addressing the dietary quality of children in Timor‐Leste would benefit from improving women's diets through better access to nutritious foods and to secondary education.

## 1 INTRODUCTION

Consuming a variety of foods is critical to meet human nutrient requirements. Dietary diversity, a key component of high‐quality diets, enables the intake of essential nutrients and promotes optimal health (Ruel, 2003). In low‐ and middle‐income countries (LMIC), lack of dietary diversity is a serious problem among groups with higher nutritional needs, such as infants and young children and women of reproductive age. Monotonous diets based on starchy staples, limited access to seasonal fruits and vegetables and occasional animal‐source foods (ASF) are associated with several micronutrient deficiencies among children (Arimond & Ruel, 2004; Moursi et al., 2008; Working Group on Infant Young Child Feeding Indicators [WGIYCFI], 2006) and women (Arimond et al., 2011; Torheim, Ferguson, Penrose, & Arimond, 2010). Moreover, ample evidence demonstrates a strong association between dietary diversity and the nutritional status of children (Arimond & Ruel, 2004; Rah et al., 2010; Sawadogo et al., 2006).

Improving the quantity and quality of foods fed to children in their first 5 years of life and those consumed by prepregnant, gestating and lactating women, is critical to reduce malnutrition in all its forms. ASF (dairy, eggs, meat, poultry and fish) are important elements of dietary quality, containing essential amino acids that regulate growth combined with micronutrients such as iron, vitamins A and B, zinc and calcium that support optimal development (Grace et al., 2018; Headey, Hirvonen, & Hoddinott, 2018). Although the relationship between ASF intake and stunting reduction during early childhood has shown mixed results (Shapiro et al., 2019), the use of ASF to mitigate micronutrient deficiencies is a widely acknowledged food‐based strategy (Leroy & Frongillo, 2007). How ASF allocation patterns differ however between children and mothers in contexts of scarcity remains poorly understood and is informed by sociocultural and household factors (Gittelsohn & Vastine, 2003). Household food security for children and women and related care practices, for example, determine child and maternal dietary intake, and when inadequate, are an immediate cause of malnutrition (United Nations Children's Fund [UNICEF], 1990). Seasonality also can mediate food availability and access in LMIC, particularly in rural areas where agriculture‐based livelihoods prevail. Yet dietary data are often collected at a single time point, lacking the ability to reflect potential changes in diets occurring through seasons and agricultural cycles (Wong et al., 2018).

In Timor‐Leste, a small post‐conflict lower middle‐income country, the prevalence of malnutrition is persistently high with 45.6% of children under 5 being stunted and 26.6% of women of reproductive age underweight, paired with anaemia levels of 40.3% and 22.7%, respectively (General Directorate of Statistics [GDS], Ministry of Health [MoH], & ICF Macro, 2018). Poor quality diets and protein deficiency in Timor‐Leste are major contributors to malnutrition (Bonis‐Profumo, McLaren, & Fanzo, 2019; Molyneux, da Cruz, Williams, Andersen, & Turner, 2012; Wong et al., 2018). High stunting levels reflect a history of war and internal displacement as well as lack of access to sufficient and nutritious foods (Reinhardt & Fanzo, 2014). Despite slow progress to improve nutritional outcomes since independence in 2002, there have been sharp decreases in child and maternal mortality rates (GDS et al., 2018) in a context where poverty affects 41.8% of the population and 70.0% live rurally (Ministry of Finance [MoF] & World Bank [WB], 2016). Some infant and young child feeding practices have improved since Timor‐Leste's sovereignty. Between 2003 and 2016, early initiation of breastfeeding increased from 47.0 to 75.0%, and the proportion of infants under 6‐months who received foods as opposed to exclusively breastfed decreased from 65.0 to 22.0% (GDS et al., 2018; MoH et al., 2004). Dietary indicators, however, remain poor. Among children 6–23 months old, 13% achieved the minimum acceptable diet in 2016, capturing both adequate diversity and meal frequency (GDS et al., 2018). Minimum dietary diversity (MDD), a proxy for micronutrient adequacy, was 33.6% for this age group (GDS et al., 2018) and 40.6% for children 24–59 months (MoH, 2015), whereas for women, nationally representative data on their achievement are unavailable. ASF intake is low, only 25.5% of children 6–23 months consumed dairy products (MoH, 2015) for example, compared with the Asian regional average, of 37.7% (Headey et al., 2018).

In a country that faces cyclical food insecurity (da Costa et al., 2013), seasonal assessments of child and maternal diets require further research. Wong, Bagnol, Grieve, Li, and Alders (2016) and Wong et al. (2018) started addressing this gap, and our work builds from it by examining agroecological considerations and a broader child age range. Localized studies have assessed smallholders' diets and identified the rainy months as the most food insecure and less diverse (da Costa et al., 2013; Glazebrook, Lopes, da Costa, & Ximenes, 2007) and higher consumption of wild foods during the dry season (Erskine et al., 2015). Most of these investigations have not measured dietary adequacy using validated indicators. Additionally, the composition of diets and access to ASF might vary according to distinct agricultural profiles due to the semisubsistence nature of Timorese rural livelihoods. Thus, the contextual determinants of dietary diversity and ASF intake among smallholders warrant exploration.

This study examines the dietary quality of children 6–59 months old and their mothers living in rural Timor‐Leste. Our aim was to first assess child and maternal dietary diversity and ASF intake differentials in two agricultural livelihood zones; second, to investigate the role of seasonality in women and children's consumption and allocation of food groups; and third, to identify household and agroecological characteristics associated with children's dietary quality in relation to their mothers'. By better understanding dietary patterns of rural‐living families and their seasonal fluctuations, policies and programmes can be better designed to improve child and maternal nutrition.

## 2 RESEARCH DESIGN AND METHODS

### 2.1 Study setting

Our research was conducted in four rural villages or *suku* of Baucau and Viqueque districts, in eastern Timor‐Leste (Figure S1). Leading income sources comprise farming and livestock rearing, and staples are maize, rice and cassava (GDS & United Nations Population Fund [UNFPA], 2016). The study *suku* belong to two agricultural livelihood zones, coastal and mid‐altitude, where more than 35% of households grow irrigated rice (Williams, Bacon, Ferreira, & Erskine, 2018; Figure S2). Coastal lowlands have lower crop‐livestock diversity and better access to markets, whereas inland mid‐altitude areas have more diverse agricultural systems and poorer roads (Williams et al., 2018). We hypothesized that mid‐altitude zones access less seafood and traded goods and that ASF consumption might be lower as a result. Out of 442 *suku* nationwide, 108 fall in these two zones, representing 29% of the population and all predominantly rice‐growing communities (Williams et al., 2018).

**FIGURE 1:**
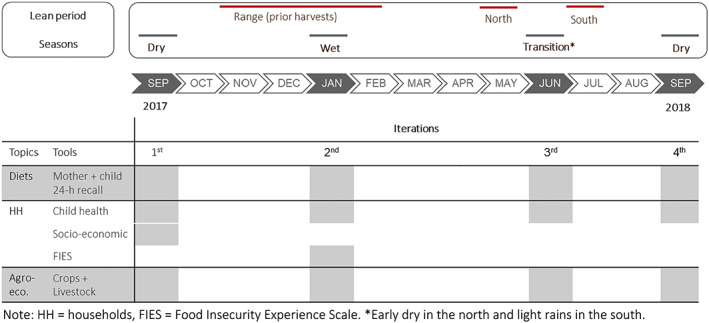
Diagram of seasons, data collection iterations and tools used

Timor‐Leste has a tropical climate with two seasons, wet and dry. The wet season ranges from November to April, with a second short rainy period between June and August in the south, followed by a dry season with virtually no rains. The lean season occurs when food reserves deplete before the maize and rice harvests (Figure 1) and is more severe in the rainfed uplands where rice is seldom grown (da Costa et al., 2013). Examined *suku* have an annual rainfall of 1,200–2,000 mm (Williams et al., 2018), making them comparable despite their disposition north and south of the island ridge (Figure S1).

### 2.2 Design and sampling

A longitudinal observational study was conducted from September 2017 to September 2018 among 200 households with a child aged 6–59 months in two coastal and two mid‐altitude *suku.* The study sites were selected through partnering with a Community Driven Nutrition Improvement Project (CDNIP), aimed to improve nutrition practices and agriculture diversification in 50 least developed *suku* in Baucau and Viqueque districts, universally targeting pregnant and lactating women with children younger than 2 years of age (WB, 2014). These districts concentrate the highest number of lowest living standard *suku* nationally (Asian Development Bank [ADB], 2013). Implemented in 2014–2018 by Catholic Relief Services, this WB‐funded intervention delivered nutrition sessions and demonstrated kitchen gardens with some material provision. In 30 *suku*, at least one ASF component was delivered as part of the CDNIP, a chicken vaccination campaign led by the Timor‐Leste Ministry of Agriculture and Fisheries, and/or fishpond support. This programme was used as an engagement platform for the study reported herein.

We estimated a sample size of 200 households equally distributed by livelihood zone to give the study 80% power to detect a conservative minimum difference in dietary diversity between zones and seasons equal to 0.5 food groups (=1/3 SD) with an *α* = 0.05. A design effect adjustment of 1.5 was included to allow for correlation among clusters (*suku*) of observations assuming an intracluster correlation equal to 0.01. An estimated 20% respondent dropout rate was added to the sample size, initially calculated at 160 households. Our convenience sampling followed multiple stages: (1) selection of CDNIP *suku* with an ASF component; (2) identification of *suku* in two livelihood zones (coastal/mid‐altitude), when multiple *suku* were eligible those with less than 50 participants were excluded and better access prioritized; and (3) random selection of 50 households per *suku.* After randomization, households were visited and invited to participate; when non‐available/uninterested, a reserve list was used until target completion. Eligible mothers had a child aged 6–48 months, lived with an adult male and were permanent residents. Youngest child, younger than 4 years, was selected, and twins were registered as one participant.

### 2.3 Data collection

Data were collected four times in 3‐ to 5‐month intervals through a tablet‐based household survey, designed in KoBoToolbox (Harvard Humanitarian Initiative, http://www.kobotoolbox.org/), and interviewer‐administered to mothers. We aimed to capture the dry (September) and wet (January) seasons, and a transition (June) period—dry in the north and light rains in the south. We measured two dry seasons to capture potential weather variations. All iterations included questions on diets (mother and child 24‐h recalls), household and agroecological (child health, crops and livestock); one included socio‐economic and food security questions, measured through the Food Insecurity Experience Scale (Figure 1).

Dietary assessments were based on the maternal recollection of all foods and beverages consumed, and given to children, 24 h before the survey. Open recall was followed by the list‐based method (Food and Agriculture Organization of the United Nations [FAO] & FHI360, 2016), where enumerators read a predefined list of foods from nonreported groups to improve completeness. Questionnaires were previously adapted to reflect locally available foods. The principal investigator, a PhD candidate, trained and accompanied the team of enumerators and reviewed data daily.

### 2.4 Variables

#### 2.4.1 Outcome variables

For children 6–23 months, we calculated the *dietary diversity score (DDS) for infant and young child feeding* by adding the number of groups consumed and MDD, as four or more food groups from seven, that is, grains/roots/tubers, legumes/nuts, dairy products (excluding sweetened milk), flesh foods (organs, meat, poultry, fish), eggs, vitamin A‐rich fruits and vegetables and other fruits and vegetables (World Health Organization [WHO], 2010). These measures include infant formula in the dairy group, penalizing breastfed children as breast milk is not counted. Despite WHO (2017) recently changing to eight‐food groups to include breast milk and remedy such incongruity, we apply the 2010 methodology to ensure comparability with children 24–59 months (as there is no age‐specific indicator). To assess low dietary diversity, we used a cut‐off of *DDS ≤ 2 food groups*, associated with poor micronutrient intake (Moursi et al., 2008).

For mothers, we measured the *DDS for women of reproductive age* (DDS‐W) and MDD (MDD‐W) achieved at five or more food groups from 10, that is, grains/white roots/tubers/plantains, pulses (beans, peas and lentils), nuts/seeds, dairy (excluding flavoured and sweetened milk), meat/poultry/fish, eggs, dark green leafy vegetables, other vitamin A‐rich fruits and vegetables, other vegetables and other fruits (FAO & FHI360, 2016).

Following the DDS, ASF were classified in three groups (dairy, flesh foods and eggs) and measured as intake of any *ASF consumed yesterday.*

#### 2.4.2 Explanatory variables

The UNICEF (1990) framework of malnutrition informed the selection of potential explanatory individual and household variables for dietary intake. Elements of agricultural systems were included as agroecological variables. To examine children's diets, maternal dietary indicators (DDS‐W, MDD‐W and ASF consumed yesterday) were included as potential explanatory factors. Individual‐level variables were child age, sex, if sick and had diarrhoea in the last 2 weeks and mother's age, education, and number of children. Household‐level measures included number of household members, sanitation status, food security and wealth index (WI) tertile. Agroecological‐level variables were cereals and vitamin A crops grown, number of buffalo and chickens, livelihood zone, north–south aspect and season.

### 2.5 Statistical analysis

Data were downloaded to MS Excel 2016 and analysed in STATA/IC 15.1, statistical significance was defined at *P* < .05, two tailed. Two indexes were built. The WI was calculated using principal component analysis of household facilities, ownership of assets and livestock, following the Demographic and Health Survey (DHS) methodology (Rutstein & Johnson, 2004). The first principal component was extracted and the WI calculated by ranking the component into tertiles weighted by the number of household members. Despite that *suku* were of lowest living standards, we aimed to rank intragroup differences. The *Food Insecurity Experience Scale* (FIES) is a dichotomous 8‐items scale assessing the conditions that reflect limited food access (FAO, 2016). We considered the individual level (mothers) for a 30‐day reference period (December 2017). Analysis applied the Rasch probabilistic model and calibrated results against a global reference scale by equating the mean and standard deviation of common severity item parameters (FAO, 2016). Data were computed in RStudio 1.2.1335 after installing the RM.weights 2.0 package to produce two equated indicators, then imported into Stata.

Descriptive statistics were used to report the frequency or continuous distribution of participants' characteristics at baseline (Table 1). To provide an overall estimation of the diets consumed by children and mothers, we calculated dietary outcomes across all four time points, as multiple recalls across seasons provide a better picture of habitual food intakes (FAO, 2018). We then analysed dietary quality indicators by livelihood zone and tested whether coastal women and children consumed more ASF‐rich diets through bivariate regressions that accounted for the repeated sampling (Table 2). Following, we explored seasonal variations in maternal and child food groups' consumption (Table 3). Finally, we modelled the relationship between child and maternal dietary indicators after controlling for potential individual, household and agroecological predictors in a multivariable mixed‐effects model using the combined dataset (Table 4).

**TABLE 1:** Distribution of household and agroecological characteristics of study participants: Overall and by livelihood zone in rural Timor‐Leste, at baseline in September 2017—dry season

Characteristics of participants (%) if unspecified	Overall	Livelihood zone	
		Coastal	Mid‐altitude
Number of mother–child dyads/households (*n*)	167	83	84
Children			
Age			
6–23 months	59.3	55.4	63.1
24–59 months	40.7	44.6	36.9
Sex			
Female	47.3	45.8	48.8
Sick in the last 2 weeks	57.5	57.8	57.1
Diarrhoea in the last 2 weeks	25.2	31.3	19.1
Among 6–23 months old			
Breastfeeding			
Fed breast milk^b^	42.4	41.3	43.4
Fed infant formula	10.1	13.0	7.6
Dietary diversity score, mean (SD)	2.5 (1.3)	2.5 (1.4)	2.5 (1.3)
Minimum dietary diversity^c^	22.2	24.5	19.6
Minimum meal frequency^d^	47.5	47.8	47.2
Minimum acceptable diet^e^	9.1	9.4	8.7
Intake of flesh foods^f^	30.3	26.1	34.0
Mothers^g^			
Dietary diversity score—women, mean (SD)	3.1 (1.3)	3.2 (1.3)	2.9 (1.3)
Minimum dietary diversity—women^h^	14.4	16.9	11.9
Age (years), mean (SD)	29.5 (7.6)	29.7 (7.7)	29.3 (7.6)
No formal education	28.1	25.3	31.0
Household ^i^			
# of members, mean (SD)	7.9 (3.1)	8.0 (3.2)	7.9 (3.0)
# of children, mean (SD)	3.7 (2.2)	3.6 (2.1)	3.8 (2.3)
Improved water source	75.9	86.6	65.5
Improved sanitation	28.1	39.8	16.7
Wealth index			
Lowest	34.1	13.3	54.8
Middle	34.7	39.8	29.8
Highest	31.1	47.0	15.5
Food Insecurity Experience Scale			
Moderate and severe^a^	22.0	19.5	25.5
Severe^a^	4.6	2.6	7.3
Income and livelihood sources^j^			
Subsistence	11.2	6.6	15.9
Crops sale	32.8	29.4	36.4
Livestock sale	29.1	22.8	35.6
Fishing	7.5	14.0	0.8
Salary	7.1	9.6	4.6
Other	12.3	17.7	6.8
Crops grown, livestock raised (%)			
Rice	58.7	51.8	65.5
Maize	55.9	41.0	66.7
Vitamin‐A fruits and vegetables	87.4	80.7	94.1
Crops, median (IQR)	4 (2–8)	3 (1–7)	6 (3–8)
Buffalo	36.5	38.1	34.9
Herd, median (IQR)	0 (0–3)	0 (0–2)	0 (0–4)
Chickens	93.4	90.4	96.4
Flock, median (IQR)	9 (4–16)	9 (4–17)	9 (5–16)
Altitude (m), median (range)^k^	307 (23–779)	82 (23–618)	412 (90–779)

Abbreviations: IQR, interquartile range; MAD, minimum acceptable diet; MDD, minimum dietary diversity—infant and young children feeding; MDD‐W, minimum dietary diversity—women of reproductive age; MMF, minimum meal frequency; SD, standard deviation.

^a^ *n* = 136 (January 2018).

^b^ Exclusive breastfeeding data not presented due to reliability issues with inclusion criteria.

^c^ MDD among children 6–23 months old. Proportion who received ≥4 food groups out of seven (WHO, 2010).

^d^ MMF among children 6–23 months old. For breastfed children, frequency of ≥2 meals for children 6–8 months and ≥3 for children 9–23 months. For non‐breastfed children, frequency of ≥4 meals, including the number of milk feeds (WHO, 2010).

^e^ MAD among children 6–23 months old. For breastfed children, proportion who met MDD and MMF. For non‐breastfed children, proportion who received ≥4 food groups out of seven excluding milk products and met the MMF plus ≥2 milk feedings (WHO, 2010).

^f^ Proportion of children 6–23 months old that received organs, meat, poultry and fish; classified as iron‐rich foods (WHO, 2010).

^g^ Three women were the child's grandmother.

^h^ MDD‐W among women aged 15–49 years. Proportion who consumed ≥5 food groups out of 10 (FAO and FHI360, 2016).

^i^ Improved water and sanitation definitions based on Census 2015 (GDS & UNFPA, 2016).

^j^ Combine primary and secondary income sources. Other includes small trade, brewing or handicraft, social assistance or remittances; salary includes waged skilled labourer, NGO employee and government staff.

^k^ Data recorded after households survey through KoboToolbox GPS feature, averaging 5.2 m in precision.

**TABLE 2:** Bivariate associations of dietary quality indicators among children 6–59 months and their mothers with livelihood zone in rural Timor‐Leste, samples across four time points 2017–2018

Dietary quality indicators	Overall	Livelihood zone				
		Coastal	Mid‐altitude	OR^a^	(95% CI)	*P* value^b^
Mother–child dyads (*n*)	167	83	84			
MDD (%)						
Children 6–23	16.0	15.5	16.5	0.78	(0.28, 2.20)	.638
Children 24–59	19.7	23.9	14.8	2.18	(0.95, 4.99)	.065
Children 6–59—All	18.3	21.0	15.5	1.51	(0.79, 2.88)	.209
DDS, mean (SD)						
Children 6–23	2.4 (1.1)	2.5 (1.2)	2.3 (1.1)	*β* 0.16	(−0.20, 0.51)	.382
Children 24–59	2.7 (1.0)	2.9 (1.1)	2.6 (1.0)	*β* 0.32	(0.05, 0.59)	.018*
Children 6–59—All	2.6 (1.1)	2.7 (1.1)	2.5 (1.0)	*β* 0.28	(0.25, 0.51)	.019*
Mothers^c^	2.7 (0.9)	2.8 (0.9)	2.5 (0.9)	*β* 0.22	(0.04, 0.41)	.016*
DDS‐W, mean (SD)^d^	2.8 (1.1)	2.9 (1.1)	2.7 (1.1)	*β* 0.21	(−0.01, 0.43)	.057
MDD‐W (%)	7.6	8.6	6.6	1.36	(0.68, 2.73)	.386
Low diversity ≤ 2 food groups (%)						
Children 6–23	56.1	50.0	61.4	0.54	(0.23, 1.24)	.145
Children 24–59	46.2	41.0	52.3	0.58	(0.34, 1.01)	.052
Children 6–59—All	50.0	44.1	56.1	0.56	(0.34, 0.90)	.016*
Mothers	45.3	40.0	50.8	0.59	(0.37, 0.94)	.027*
ASF groups yesterday (%)^e^						
Children 6–23	40.5	44.6	37.0	1.53	(0.73, 3.23)	.264
Children 24–59	48.8	56.1	40.3	2.19	(1.23, 3.88)	.007**
Children 6–59—All	45.6	52.1	38.9	1.90	(1.18, 3.07)	.008**
Mothers	37.5	42.9	31.3	1.54	(0.98, 2.44)	.064

*Note*: The table presents outcomes for samples across four time points, totalling 1,236 dietary recalls from 167 mother–child dyads: 618 for mothers, 381 for children aged 24–59 months, and 237 for children 6–23 months old. Frequencies and means are across seasons, to be interpreted as incidences of dietary recalls. Modelling based on mixed‐effects GLMM with a random intercept (household) accounting for repeated sampling. Accordingly, odds ratios and coefficient estimates account for multiple observations over time, to be interpreted at the participant level.

Abbreviations: ASF, animal‐source foods; DDS, dietary diversity score—infant and young children feeding; DDS‐W, dietary diversity score—women of reproductive age; GLMM, generalized linear mixed models; MDD, minimum dietary diversity—infant and young children feeding; MDD‐W, minimum dietary diversity—women of reproductive age; SD, standard deviation.

^a^ OR = odds ratio, unless *β* = coefficient if specified. Mid‐altitude zone is the reference value. CI, confidence interval.

^b^ *P* values are a test of association between participants' dietary quality indicator and livelihood zone, applying a mixed‐effects logistic model for binary outcomes (OR) and a generalized linear mixed‐effects model for continuous outcomes (*β*).

^c^ To enable mothers' and child DDS comparability, the seven‐food groups indicator is presented for mothers.

^d^ DDS and DDS‐W cannot be compared as they use different food grouping parameters.

^e^ ASF groups: dairy products (excluding sweet condensed milk), flesh foods (organs, red meat, poultry and fish/shellfish) and eggs.

* *P* < .05.

** *P* < .01.

*** *P* < .001.

**TABLE 3:** Seasonal food groups and animal‐source foods consumption and dietary diversity scores of women and children 6–59 months old in rural Timor‐Leste, samples across four time points 2017–2018

Seasonal dietary diversity indicators	Seasons				
	Dry	Wet	Transition^a^	Dry	*P* value^b^
	Sep 2017	Jan 2018	Jun 2018	Sep 2018	
Women (*n*)	167	136^c^	158	157	
DDS‐W food groups (%)					
Grains, white roots, tuber and plantain	100	100	100	100	‐
Pulses (beans, peas and lentils)	8	8	8	6	.754
Nuts and seeds	5	4	0	1	.153
Dairy	3	4	1	0	.218
Meat, poultry and fish	41	31	23	43	<.001***
*Organs*	1	2	1	2	.675
*Red meat*	26	10	13	27	<.001***
*Poultry*	4	5	5	4	.846
*Fish*	15	20	8	15	.019*
Eggs	5	4	6	3	.632
Dark green leafy vegetables	74	84	77	60	<.001***
Other vitamin A‐rich fruits and vegetables	23	9	22	10	<.001***
Other vegetables	37	34	33	33	.847
Other fruits	13	10	9	4	.036*
DDS‐W, mean (SD)	3.1 (1.3)	2.9 (1.0)	2.8 (1.0)	2.6 (1.0)	<.001***
Low diversity (≤2 FG) (%)	38.3	39.7	51.9	51.0	.009**
Children 6–59 months (*n*)	167	136^c^	158	157	
DDS food groups (%)					
Grains, roots and tubers	98	100	100	99	‐
Legumes and nuts	10	7	7	4	.149
Dairy products	21	14	13	3	<.001***
Flesh foods	38	31	21	41	<.001***
*Organ meat*	1	2	1	1	.576
*Red meat*	25	10	13	24	.001**
*Poultry*	2	7	5	5	.185
*Fish*	14	17	6	14	.032*
Eggs	14	17	8	6	.005**
Vitamin A‐rich fruits and vegetables	68	68	75	63	.086
Other fruits and vegetables	28	29	29	30	.984
DDS, mean (SD)	2.8 (1.3)	2.7 (1.1)	2.5 (0.9)	2.5 (1.0)	.012*
Low diversity (≤2 FG) (%)	44.9	46.3	54.4	54.1	.132

*Note*: ASF are highlighted in grey, with flesh foods disaggregated in four categories in italics.

Abbreviations: ASF, animal‐source foods; DDS, dietary diversity score—infant and young child feeding; DDS‐W, dietary diversity score—women of reproductive age; FG, food groups; SD, standard deviation.

^a^ Early dry in the north and late wet in the south.

^b^ *P* value is for the Walt test performed after (i) mixed‐effects logistic model of association between the percentage of participants consuming a food group and season and (ii) mixed‐effects linear model of association between mean DDS‐W or DDS and season.

^c^ In January 2018, 25 remote households were not interviewed due to accessibility limitations during the heavy rains period.

* *P* < .05.

** *P* < .01.

*** *P* < .001.

**TABLE 4:** Characteristics associated with dietary quality outcomes among children 6–59 months old in rural Timor‐Leste, samples across four time points 2017–2018

Predictor variables Mother–child dyads (*n* = 167)	Child dietary quality outcomes											
	DDS^a^				MDD^b^				ASF consumed yesterday^b^			
	Bivariate		Multivariable		Bivariate		Multivariable		Bivariate		Multivariable	
	*β*	(95% CI)	*β*	(95% CI)	OR	(95% CI)	OR	(95% CI)	OR	95% CI	OR	95% CI
DDS‐W^c^	0.55***	(0.49, 0.62)	0.40***	(0.34, 0.46)	4.07***	(2.95, 5.61)			2.43***	(1.93, 3.06)		
MDD‐W^c^												
No	Reference											
Yes	1.11***	(0.82, 1.39)			17.82***	(7.45, 42.63)	9.80***	(3.81, 25.23)	5.43***	(2.36, 12.47)		
ASF yesterday												
No	Reference											
Yes	0.82***	(0.66, 0.97)			6.48***	(3.70, 11.32)			29.63***	(16.78, 52.34)	21.83***	(12.46, 38.26)
Age group^d^												
6–23 months	Reference											
24–59 months	0.07	(−0.12, 0.25)	0.20**	(0.07, 0.32)	0.94	(0.52, 1.73)			1.19	(0.77, 1.85)		
MDD												
No	Reference											
Yes	2.05***	(1.90, 2.19)							25.16***	(11.66, 54.29)	15.51***	(6.97, 34.50)
ASF yesterday												
No	Reference											
Yes	1.25***	(1.12, 1.38)	0.94***	(0.81, 1.06)	25.58***	(11.61, 56.35)	22.77***	(9.77, 53.05)				
Education												
No schooling	Reference											
Primary	0.36*	(0.07, 0.64)	0.20*	(0.04, 0.38)	2.85*	(1.18, 6.86)	2.37	(0.92, 6.12)	1.06	(0.59, 1.93)		
Secondary or +	0.57***	(0.29, 0.85)	0.21*	(0.04, 0.39)	5.85***	(2.50, 13.67)	4.09**	(1.67, 9.98)	2.28**	(1.26, 4.11)		
Number members												
3–7	Reference											
>7	−0.30*	(−0.53, −0.07)	−0.12	(−0.26, 0.01)	0.60	(0.32, 1.16)			0.52***	(0.32, 0.83)	0.57*	(0.35, 0.92)
Sanitation												
Unimproved	Reference											
Improved	0.51***	(0.26, 0.76)	0.16*	(0.01, 0.26)	3.07**	(1.55, 6.10)			2.00*	(1,17, 3.41)		
Wealth index												
Lowest	Reference											
Middle	0.05	(−0.23, 0.33)			1.12	(0.53, 2.63)			1.10	(0.62, 1.96)		
Highest	0.38**	(0.10, 0.67)			2.17	(0.98, 4.81)			1.61	(0.89, 2.94)		
Livelihood zone^e^												
Mid‐altitude	Reference											
Coastal	0.28*	(0.04, 0.51)			1.51	(0.79, 2.89)			1.90**	(1.18, 3.07)		
Aspect												
North	Reference											
South	0.39**	(0.16, 0.62)	0.12	(−0.01, 0.26)	2.28*	(1.20, 4.33)			1.81*	(1.12, 2.91)		
Season												
Dry	Reference											
Wet	−0.10	(−0.31, 0.10)			0.36**	(0.19, 0.71)	0.40*	(0.19, 0.87)	0.78	(0.48, 1.30)		
Transition	−0.24*	(−0.44, −0.05)			0.27***	(0.14, 0.52)	0.42*	(0.19, 0.92)	0.44**	(0.26, 0.72)		
Dry	−0.30**	(−0.49, −0.10)			0.21***	(0.10, 0.42)	0.25**	(0.11, 0.58)	0.64	(0.39, 1.04)		

*Note*: The table analyses the sample across four time points, totalling 618 dietary recalls for children 6–59 months old and 618 dietary recalls for mothers. *β* = coefficient. Variables with a significant bivariate association (*P* < .05) are shown in the table and included in multivariable models as a fixed effect. The final model for each outcome was built by excluding non‐significant variables (*P* > .10) through backward stepwise selection of predictors.

Abbreviations: CI, confidence interval; ASF, animal‐source foods; DDS, dietary diversity score—infant and young children feeding; DDS‐W, dietary diversity score—women of reproductive age; MDD, minimum dietary diversity—infant and young children feeding; MDD‐W, minimum dietary diversity—women of reproductive age; OR, odds ratio.

^a^ Results from generalized linear mixed models (GLMM) accounting for households and sampling rounds.

^b^ Results from mixed‐effects logistic model accounting for households and sampling rounds.

^c^ For correlated predictors (DDS and MDD), only one was chosen per model and participant.

^d^ Child age group was kept in the multivariable models regardless of its significance due to being a child characteristic of interest in this study.

^e^ Livelihood zone and aspect aggregate *suku* in distinct pairs, used as fixed factors in the models instead of *suku.*

* *P* < .05.

** *P* < .01.

*** *P* < .001.

Given the longitudinal nature of the data, we used generalized linear mixed models (GLMM) in all regressions. These models included a random intercept to account for the autocorrelation of observations collected from the same subject over time and clustering of the observations from the same household, incorporating both random and fixed effects. We applied the logistic form of GLMM for binary outcomes. To build the final models, we first performed bivariate GLMM analyses to test associations between children's outcomes (DDS, MDD and ASF consumed yesterday) and potential explanatory predictors (Table S4). Covariates with a significant bivariate association (*P* < .05) were included in the initial multivariable regression models as a fixed effect. For each outcome, the final model was built by excluding non‐significant variables (*P* > .10) through backward stepwise selection of predictors. We included one dietary diversity indicator per model, due to collinearity as tested using the variance inflation factor (*collin* command), and two fixed factors, livelihood zone and aspect, which aggregated *suku* in pairs. Model residual diagnostics were performed for final variables to determine impact of potential outliers on model coefficients. Residual plots across explanatory variables indicated a random residual spread.

### 2.6 Ethical considerations

Ethical approval was provided by the Menzies School of Health Research (Ref: 2016–2719) and the National Health Institute in Timor‐Leste (Ref: 41/MS‐INS/DE‐DP/GBP/I/2017). Women provided written consent or fingerprint after verbal explanation of the study and its voluntary nature and received vegetable seeds and a family photograph as gift.

## 3 RESULTS

The final sample included 167 mother–child dyads with 618 respective dietary recalls across the seasons, totalling 1,236 observations over 12 months. Thirty‐three dyads (16.5%) were excluded due to having less than three observations, we assumed that participants' unavailability did not correspond with specific characteristics and found no major differences (Table S1). Dyads were evenly distributed across livelihood zones.

At baseline, 59.3% of children were younger than 2 years old (*n* = 99), 21.0% 6–11 months and 38.3% 12–23 months, whereas 40.7% were aged 2–5 years (*n* = 68). Among children 6–23 months, 22.2% achieved MDD, higher among those from coastal (24.5%) than mid‐altitude (19.6%) livelihood zones, whereas the DDS was equally distributed (2.5 ± 1.3; Table 1). Overall, only 9.1% of young children achieved the minimum acceptable diet and 30.3% consumed flesh foods, whereas 25.2% of all children had diarrhoea in the previous weeks. Women's DDS was 3.1 ± 1.3, and 14.4% achieved MDD‐W. Over one‐quarter of mothers were unschooled, 22.0% experienced moderate to severe food insecurity and 28.1% accessed improved sanitation. Mid‐altitude households had a higher proportion of lowest wealth tertile (54.8%) whereas coastal zones had a greater percentage of highest wealth tertile households (47.0%), as well as lower crop diversity.

### 3.1 Dietary quality indicators by livelihood zone

On aggregating dietary recalls across the four data points, we found that almost all child and maternal dietary indicators scored higher in quality in coastal zones, yet only some associations were statistically significant (Table 2). The DDS derived from aggregated recalls for coastal children 24–59 months, whose mean was 2.9 ± 1.1, was a third of a food group higher than in mid‐altitude areas (95% CI: 0.05, 0.59; *P* = .018). This pattern was also observed for children 6–59 months (*β* = 0.28; *P* = .019) but not for the younger cohort, who overall consumed less diverse diets. Although the dietary recalls of older children were 2.18 times more likely to report four or more groups in coastal areas, this difference was not statistically significant (*P* = .065). More recalls collected for children achieved MDD at least once in the coast, 45.8%, compared with 40.5% in mid‐altitude zones, whereas for mothers the ratio was much lower, 27.7% and 20.2%, respectively (Figure S3). An extremely low proportion of maternal dietary recalls (less than 10%) met MDD‐W. In mid‐altitude zones, recalls of children and mothers were more likely to show two or less food groups (*P* < .030), an indicator of poor micronutrient intake.

A larger proportion of children's dietary recalls (45.6%) included ASF than women's (37.5%), consistent across zones. Overall, recalls among children 24–59 months reported more dairy products and eggs and slightly more red meat and poultry compared with their mothers'; whereas those from children 6–23 months recorded more dairy and eggs (Figure S4). Recalls of coastal children were 1.90 times more likely to include ASF in the last 24‐h (95% CI: 1.18, 3.07; *P* = .008). Data on frequency intake of ASF types (Table S2) showed significantly higher coefficients for dietary recalls among coastal children and women (*β* = 0.48, *P* < .010). More maternal recalls did not include any ASF in the past week (17.5%) compared with those for older children (13.1%). In coastal zones, recalls of children had at least twice greater odds to include eggs (OR = 2.11; *P* = .034), fish (OR = 2.56; *P* < .001) and dairy (OR = 2.95; *P* < .001), whereas the likelihood of including red meat was 33% lower (OR = 0.67; *P* = .063) than that of mid‐altitude children.

### 3.2 Seasonal variation of diets

Dietary composition was seasonal, particularly ASF intake among children, and specific ASF and most vegetables and fruits among mothers (Table 3). Flesh foods showed a notable seasonal trend with greater consumption in the dry seasons when around 40% of women and children consumed flesh foods, highly significant for both (*P* < .001). Child and maternal intake of red meat halved during the wet and transition seasons (10.3–13.3%). The odds of children consuming flesh foods were lowest in the transition period (June), 0.36 (95% CI: 0.21, 0.63; *P* < .001) times or 64% lower than in the first dry season (data not shown). Fish consumption was associated with seasonality, highest in the wet (16.9–19.9%) and lowest in June (6.3–7.6%), whereas poultry intake was low (less than 7%). Dairy product consumption was marginal for mothers (less than 3.7%), whereas among children, intake declined sharply from 21.0% to 2.6% (*P* < .001). Children's egg consumption was around double that of their mothers and highest in the wet (16.9%; *P* = .005). Maternal intake of dark green leafy vegetables topped in the wet (83.8%) and dropped in the second dry season (59.9%; *P* < .001), also found for other vitamin A‐rich fruits and vegetables, and other fruits (*P* = .036). Children's intake of fruits and vegetables was independent of season.

These patterns were found similarly in northern and southern *suku* (Table S3). Nonetheless, as children grew older in age, their mean food groups' consumption increased gradually (Figure S5). DDS did not show a distinctive reduction in the wet season, as expected.

### 3.3 Characteristics associated with child dietary diversity and ASF intake

After controlling for household and agroecological factors, presented for each model, we found that maternal dietary indicators were associated with all three children's dietary outcomes with a high level of confidence (*P* < .001; Table 4), revealing that what mothers eat is a sound predictor of children's dietary quality among rural communities in Timor‐Leste. First, child and maternal DDS were positively and strongly associated. For every increase in a mother's food group intake, the child's DDS increased close to half a food group (*β* = 0.40; 95% CI: 0.34, 0.46; *P* < .001). Moreover, being 24–59 months old (*β* = 0.20; *P* = .002), having consumed an ASF group the day before (*β* = 0.94; *P* < .001), with a mother that attended primary school (*β* = 0.20; *P* = .018) or secondary or above (*β* = 0.21; *P* = .017), and access to improved sanitation (*β* = 0.16; *P* = .037), were significantly associated with higher DDS. Second, the odds of achieving MDD were 9.80 times higher among children whose mother achieved MDD‐W (*P* < .001). Significant predictors controlled for were child consumption of ASF (OR = 22.77; 95% CI: 9.77, 53.05; *P* < .001), mother's secondary education or more (OR = 4.09; *P* = .002) and season through a negative association and lowest in the second dry season (OR = 0.25; *P* < .001). The predictor with the largest effect size for a child achieving MDD was consuming an ASF the day before. Third, mother's intake of ASF was strongly associated with a child also consuming ASF (OR = 21.83; *P* = .001). Children were significantly less likely to consume an ASF the day before when living in a household with more than seven members, lowering their odds by 43% (OR = 0.57; *P* = .021). Ownership of buffalo or chickens were not associated with ASF intake (Table S4). Final models included WI, north–south aspect and livelihood zone as predictors, which despite statistically significant in the bivariate analyses, these lost significance after controlling for other covariates.

## 4 DISCUSSION

This study examined child and maternal dietary quality across the seasons in rural Timor‐Leste and provides a novel depiction of dietary diversity and animal‐sourced foods consumption in coastal and mid‐altitude livelihood zones. We found the dietary indicators of mothers to be strong predictors of children's dietary quality achievements after controlling for individual, household and agroecological characteristics. Mean food group intake of women and children 24–59 months were almost identical, suggesting that when foods are consumed by mothers, these are also given to older children. Diets for children 6–23 months were consistently less varied. Children whose mothers ate more food groups and consumed ASF the day before had higher odds to receive a more diverse diet. Thus, the composition of mothers' meals is likely to be an important factor in children's dietary quality. Overall, however, mothers consumed diets were of very poor quality. The importance of diets for improved nutritional status in Timor‐Leste is highlighted by a 2013 nationally representative survey that found significant associations between more frequent consumption of vitamin A‐rich fruits and eggs in households with lower maternal undernutrition and between adequate dietary practices and lower prevalence of both stunting and anaemia among children younger than 5 (MoH, 2015).

Child and maternal DDSs showed significant yet small seasonal variations, lower in the transition and 2018 dry periods. Accordingly, season was significantly and negatively associated with child MDD in the multivariable model. The intake of ASF was distinctively associated with season for both mothers and children, who were much less likely to consume flesh foods and red meat outside of the dry season. Such pattern is aligned with sociocultural practices in rural Timor‐Leste where the most common occasion to consume ASF is during cultural ceremonies, concentrated in the dry season (Wong et al., 2018, 2016). During the transition season, ASF were least consumed and low dietary diversity was highest, indicating greater child and maternal nutritional vulnerability in June. This finding differs from the typical lean period in the wet season (da Costa et al., 2013), yet corresponds with the rice pre‐harvest season among northern *suku*, suggesting distinct nutrition insecurity patterns in rice‐farming zones. Women's intake of fruits and vegetables was negatively associated with season and showed lower consumption in the dry period of 2018 compared with 2017, coinciding with drought conditions in southern *suku* (FAO, 2019).

As expected, the intake of ASF was a sound predictor of DDS and MDD. More children consumed an ASF group the day before compared with their mothers, independent from age group. For instance, a higher proportion of children received eggs and dairy compared with mothers, as Wong et al. (2018) also found, suggesting that when these nutrient‐dense foods are accessible, mothers prioritize them to children. Hence, women's selection and preferential allocation of ASF indicates animal foods are perceived as adequate for children. Dairy intake showed a negative seasonal trend, which could reflect a decrease in the disposable income of households during this period because dairy products in Timor‐Leste are purchased due to ruminants not being customarily milked. Political instability in 2018 could have triggered money‐saving behaviours.

Previous studies have recorded a positive relationship between maternal and child diets similar to ours. A report analysing 2009/2010 and 2016 DHS Timor‐Leste data found that mothers' achievement of MDD was lower than for young children when using the seven‐food group indicator (University Research Co., 2018). In contrast, in rural Tajikistan, MDD‐W was largely achieved by mothers while less than half of children 6–23 months met MDD, despite child and maternal DDS were positively associated (Klassen et al., 2019), analogous to our findings. Research among U.K. dyads concluded that the quality of mothers' diets was the leading explanatory factor informing children's dietary quality (Fisk et al., 2011), highlighting that shared family patterns are also expressed in resource‐abundant settings. Regarding the role of seasonality, a study in Zambia observed children's diets to be more diverse during the early lean season (Caswell, Talegawkar, Siamusantu, West, & Palmer, 2018), contrary to expectations and equivalent to our results. In terms of ASF, in Tanzania, a study showed that the frequency of children's poultry products consumption was predicted by their mother's intake (de Bruyn et al., 2018), further exemplifying the linkages between child and maternal dietary patterns.

Additionally, we found mother's schooling, particularly secondary achievement, to be positively associated with both diversity indicators for children. Access to improved sanitation was significant with DDS, and residing with less than seven household members increased the odds of children consuming ASF. Agroecological and socio‐economic factors played a smaller role. In LMIC, maternal education is a prevailing determinant of children's dietary diversity (Blackstone & Sanghvi, 2018; Choudhury, Headey, & Masters, 2019; Senarath et al., 2012), whereas agroecological factors show context‐specific results (Choudhury et al., 2019). Moreover, a study in neighbouring Indonesia found that determinants of young children's DDS and ASF intake included mother's knowledge—combining education level and media access, as well as socio‐economic status (Sebayang et al., 2019). Yet we found the intragroup WI to be non‐significant, probably due to included *suku* falling in the lowest national living standards (ADB, 2013). Our findings suggest that mother's access to nutritious foods and education contribute most to children's dietary outcomes in rural Timor‐Leste.

The quality of diets differed by livelihood zone. In coastal areas, almost all child and maternal indicators scored higher when compared with mid‐altitude zones, suggesting access to slightly better‐quality meals. However, the zone effect lost its significance in the final model when controlled for other covariates. This could be due to the living standards homogeneity of *suku* involved, which might impact on the explanatory power of agroecological parameters. The findings are aligned with those from previous studies in Timor‐Leste. For example, coastal households had higher DDS than those from mountainous villages (3.03 ± 0.88 vs. 2.68 ± 0.79), yet caution should be applied when comparing with this study due to differences in measurements (Spencer, Sanders, & Judge, 2018). Our results found higher odds to consume eggs, fish and dairy products among coastal children, whereas red meat was more prevalent in mid‐altitude zones. Surprisingly, livestock ownership was not statistically associated with children's ASF consumption, perhaps due to the importance of livestock for income, rituals, and status (Bettencourt, Tilman, Narciso, Carvalho, & Henriques, 2015). Lesser ASF intake in mid‐altitude *suku* could reflect lower market access and asset levels—a proxy for household income, as described in the zones characterization (Williams et al., 2018).

### 4.1 Strengths, limitations and implications

Key strengths of this study were the volume of dietary recalls obtained over 1 year and data collection quality through the recruitment of local and trained enumerators. We faced several limitations. The longitudinal design incurred participant dropout, which we assumed to be independent of specific characteristics, yet we lack certainty. Our sample size and geography were not representative of all rural populations, limiting the results' generalizability. Dietary data were self‐reported, thus prone to recall error. It is plausible that foods recommended by the intervention, including orange‐flesh sweet potatoes, moringa (*Moringa oleifera*) and eggs, were overreported due to participant bias and socially desirable responses. This bias would be consistent across *suku.*

Our results show that policies and programmes aimed at improving infant and young child feeding practices in Timor‐Leste would gain from supporting mother's access to nutrient‐dense foods and secondary education. Nutrition‐sensitive agriculture programmes could have an important role to play to improve the production of nutritious foods, including ASF, particularly in inland areas. Smallholders would benefit from widespread market access to affordable eggs and non‐sugary dairy products. Efforts are required to continue promoting optimal feeding practices for children 6–23 months, more nutritionally at risk. Future research could focus on exploring the role of decision making over what foods are consumed within households, as well as distribution patterns among family members. Moreover, because most smallholders own livestock, notably chickens and pigs (GDS & UNFPA, 2016), the relationship between livestock ownership and ASF intake requires further investigation.

## 5 CONCLUSION

In this study, we investigated the dietary quality of mothers and children 6–59 months old in rural Timor‐Leste and explored agroecological factors including seasonality. We found that, first, dietary diversity and ASF intake were marginally better in coastal livelihood zones. Yet women's dietary achievements were outstandingly poor—only a fraction was likely to meet micronutrient adequacy, and their consumption of ASF was lower than among children. Second, that seasonality predicted the intake of flesh foods and red meat, both much more likely to be consumed during the dry season. Lower quality diets were more acute during the transition season in June, suggesting distinct nutrition insecurity patterns. Further, women exhibited preferential allocation patterns of specific ASF to children, dairy products and eggs. Third, we found a positive and strongly significant association between children's dietary indicators, DDS, MDD and ASF consumption, and those of their mothers'. Maternal dietary quality and education achievement, more so than agroecological characteristics, were explanatory factors of children's diet among our sample. These findings contribute to the understanding of rural diets' contextual determinants in LMIC and provide insights on ASF allocation patterns in a unique sociocultural setting. Overall, our study highlights that addressing the dietary quality of children in Timor‐Leste, as has been shown in other resource‐poor settings, would benefit from improving women's diets through better access to nutritious foods and to secondary education.

## CONFLICTS OF INTEREST

The authors declare that they have no conflicts of interest.

## CONTRIBUTIONS

GBP conceptualized the study; NS and JB provided input into the research design; GBP coordinated data collection and conducted data analysis; JB provided support for quantitative analyses; GBP wrote the initial and subsequent drafts of the manuscript; NS and JB contributed to critical revisions of the manuscript.

## Supporting information


**Figure S1.** Map with research sites and annual rainfall (mm)
**Figure S2.** Map with research sites and agricultural livelihood zones: North and South coast irrigated areas (coastal) and Mid‐altitude irrigated areas (mid‐altitude)
**Table S1.** Key characteristics and indicators of the initial sample and included participants in the final analysis at baseline in rural Timor‐Leste, September 2017
**Figure S3.** Number of instances individual children and women achieved Minimum Dietary Diversity (MDD), in rural Timor‐Leste, samples across 4 time points 2017–2018
**Figure S4.** Food groups (A) and animal‐source foods types (B) consumed over a 24‐h period by children (6–23 and 24–59 months) and their mothers (*n* = 167 dyads) in rural Timor‐Leste, samples across 4 time points 2017–2018
**Table S2.** Bivariate associations between animal‐source foods consumed last week among children 6–59 months and mothers with livelihood zone in rural Timor‐Leste, samples across 4 time points 2017–2018
**Table S3.** Seasonal food groups and animal‐source foods consumption and dietary diversity scores of women and children 6–59 months old by north–south aspect in rural Timor‐Leste, samples across 4 time points 2017–2018
**Figure S5.** Mean (standard error) food groups consumed yesterday among children by age group, comprising 618 dietary recalls across the seasons in both livelihood zones, samples across 4 time points 2017–2018
**Table S4.** Bivariate analyses with each pre‐selected covariates for the three outcome variables of child dietary quality, i.e. DDS, MDD and ASF consumed yesterday, samples across 4 time points 2017–2018
