# Supplementary material for: Maternal diets matter for children's dietary quality: Seasonal dietary diversity and animal‐source foods consumption in rural Timor‐Leste
Source: Matern Child Nutr. 2020 Aug 5;17(1):e13071. doi: 10.1111/mcn.13071 (PMC7729527; doi:10.1111/mcn.13071)
Supplement: Supplementary file 1 — Figure S1. Map with research sites and annual rainfall (mm) Figure S2. Map with research sites and agricultural livelihood zones: North and South coast irrigated areas (coastal) and Mid‐altitude irrigated areas (mid‐altitude) Table S1. Key characteristics and indicators of the initial sample and included participants in the final analysis at baseline in rural Timor‐Leste, September 2017 Figure S3. Number of instances individual children and women achieved Minimum Dietary Diversity (MDD), in rural Timor‐Leste, samples across 4 time points 2017–2018 Figure S4. Food groups (A) and animal‐source foods types (B) consumed over a 24‐h period by children (6–23 and 24–59 months) and their mothers (n = 167 dyads) in rural Timor‐Leste, samples across 4 time points 2017–2018 Table S2. Bivariate associations between animal‐source foods consumed last week among children 6–59 months and mothers with livelihood zone in rural Timor‐Leste, samples across 4 time points 2017–2018 Table S3. Seasonal food groups and animal‐source foods consumption and dietary diversity scores of women and children 6–59 months old by north–south aspect in rural Timor‐Leste, samples across 4 time points 2017–2018 Figure S5. Mean (standard error) food groups consumed yesterday among children by age group, comprising 618 dietary recalls across the seasons in both livelihood zones, samples across 4 time points 2017–2018 Table S4. Bivariate analyses with each pre‐selected covariates for the three outcome variables of child dietary quality, i.e. DDS, MDD and ASF consumed yesterday, samples across 4 time points 2017–2018 [file MCN-17-e13071-s001.docx]

**Supporting information**

Figure S1 – Map with research sites and annual rainfall (mm)


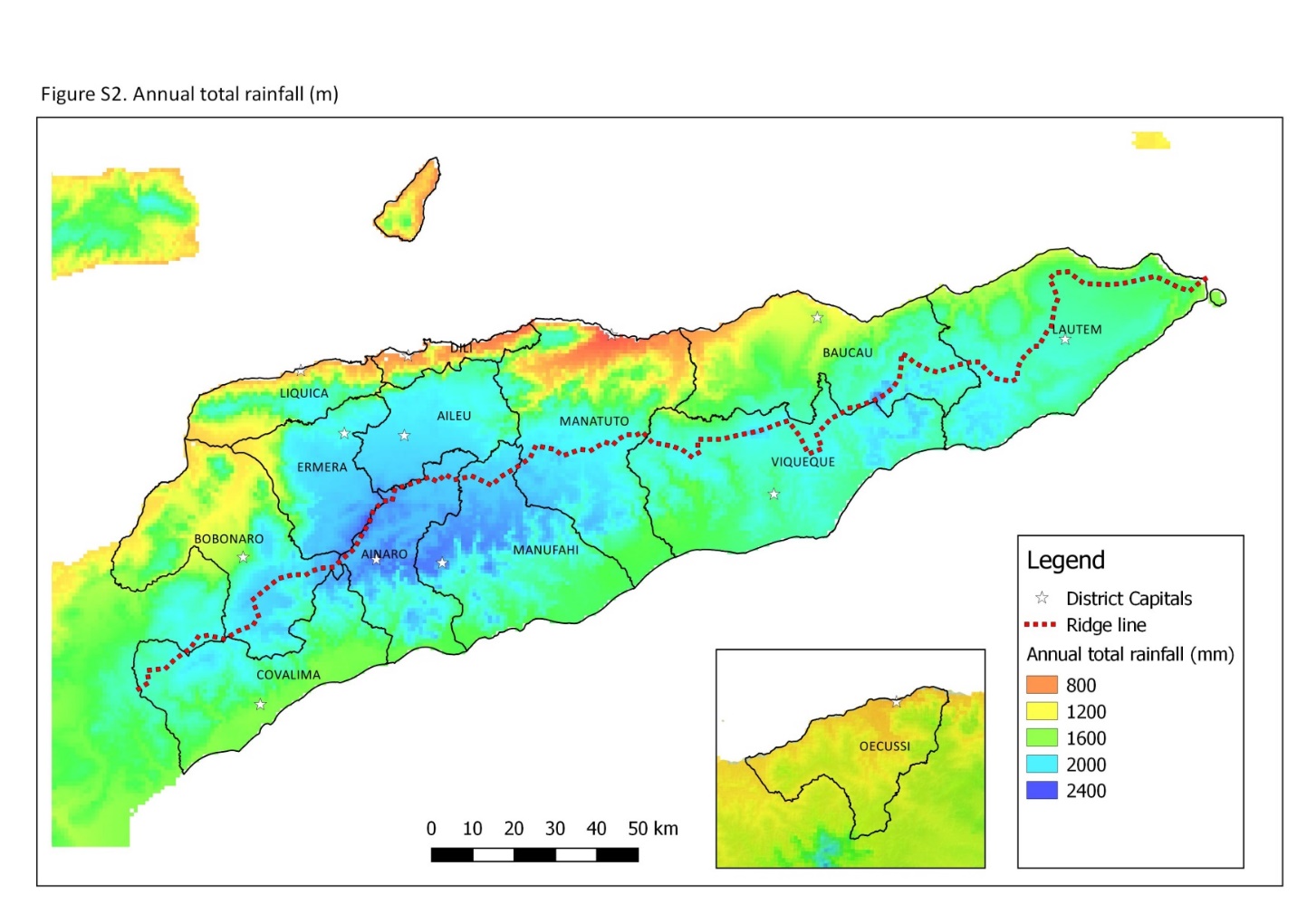
Note: Rainfall in Baucau *suku* range from 1200 to 2000mm, in Viqueque *suku* from 1600 to 2000mm.

Source: Williams et al., 2018. Figure S2 in supplementary materials.

Figure S2 – Map with research sites and agricultural livelihood zones: North and South coast irrigated areas (coastal) and Mid altitude irrigated areas (mid-altitude)

Note: The North coastal *suku* changed its typology to ‘North coast irrigated areas’ when using Census data 2015 (Williams, R. 2017. Livelihood Zone Update Using 2015 census data -unpublished), as opposed to the above map with 2010 Census data.

Source: Williams et al., 2018:865.

Table S1 – Key characteristics and indicators of the initial sample and included participants in the final analysis at baseline in rural Timor-Leste, September 2017

| Characteristics of participants  (%) if unspecified | Baseline participants | |
| --- | --- | --- |
|  | **Initial sample** | **Included in analysis** |
| Number of mother-child dyads / households (n) | 200 | 167 |
| Children 6-59 months |  |  |
| Age: 6-23 months | 61.0 | 59.3 |
| Sex: female | 49.5 | 47.3 |
| Dietary Diversity Score, mean (SD) | 2.7 (1.3) | 2.7 (1.3) |
| Minimum Dietary Diversity | 28.0 | 27.5 |
| Intake of ASF yesterday | 46.0 | 46.7 |
| Mothers |  |  |
| Dietary Diversity Score - Women, mean (SD) | 3.2 (1.3) | 3.1 (1.3) |
| Minimum Dietary Diversity - Women | 16.0 | 14.4 |
| Intake of ASF yesterday | 41.5 | 42.5 |
| Age (years), mean (SD) | 29.4 (7.7) | 29.5 (7.6) |
| No formal education | 27.5 | 28.1 |
| Household |  |  |
| # of members, mean (SD) | 7.9 (3.0) | 7.9 (3.1) |
| Improved sanitation | 29.0 | 28.1 |
| Agro-ecological |  |  |
| Livelihood zone: Coastal | 50.0 | 49.7 |
| Mid-altitude | 50.0 | 50.3 |

Note. The baseline survey collected data from 200 households. Only participants with ≥3 data points out of four were included in the final analysis (n=167).

Figure S3 – Number of instances individual children and women achieved Minimum Dietary Diversity (MDD), in rural Timor-Leste, samples across 4 time points 2017-2018

Note. MDD = minimum dietary diversity –infant and young children feeding. MDD-W = minimum dietary diversity –women of reproductive age.

The table analyses the sample across four time points, totalling 618 dietary recalls for children 6-59 months old and 618 dietary recalls for mothers.

Figure S4 – Food groups (A) and animal-source foods types (B) consumed over a 24-h period by children (6-23 and 24-59 months) and their mothers (n=167 dyads) in rural Timor-Leste, samples across 4 time points 2017-2018

A

Note. Dietary Diversity Score –Infant and Young Child Feeding (DDS) includes 7-food groups: Grains, roots, and tubers (disaggregated to illustrate different introduction patterns); Legumes and nuts; Dairy products (excluding sweet condensed milk); Flesh foods (organs, red meat, poultry, fish); Eggs; Vitamin A-rich fruits and vegetables; Other fruits and vegetables.

The table presents data for samples across 4 time points, totaling 1,236 dietary recalls: 618 for mothers, 381 for children aged 24-59 months, and 237 for children 6-23 months old.

Table S2 – Bivariate associations between animal-source foods consumed last week among children 6-59 months and mothers with livelihood zone in rural Timor-Leste, samples across 4 time points 2017-2018

| **Animal-Source Foods (ASF)**^1^  **consumed last week** | **Overall** | **Livelihood zone** | | |
| --- | --- | --- | --- | --- |
|  |  | **Coastal** | **Mid-altitude** | **OR^2^ (95% CI) *P*-value^3^** |
| Mother-child dyads (n) | 167 | 83 | 84 |  |
| **ASF types last week, mean (SD)** | | | | |
| Children 6-23 | 2.2 (1.5) | 2.4 (1.7) | 1.9 (1.4) | β 0.49 (0.01, 0.97) .048* |
| Children 24-59 | 2.5 (1.5) | 2.7 (1.6) | 2.3 (1.4) | β 0.47 (0.05, 0.88) .029* |
| Children 6-59 - All | 2.4 (1.5) | 2.6 (1.6) | 2.1 (1.4) | β 0.48 (0.14, 0.84) .006** |
| Mothers | 2.1 (1.5) | 2.4 (1.6) | 1.9 (1.4) | β 0.48 (0.15, 0.81) .004** |
| **No ASF last week (%)** | | | | |
| Children 6-23 | 19.0 | 19.1 | 18.9 | 1.03 (0.47, 2.27) .932 |
| Children 24-59 | 13.1 | 10.2 | 16.5 | 0.51 (0.20, 1.31) .164 |
| Children 6-59 - All | 15.4 | 13.3 | 17.5 | 0.65 (0.33, 1.29) .217 |
| Mothers | 17.5 | 15.6 | 19.5 | 0.68 (0.36, 1.29) .235 |
| **Types of ASF eaten at least once last week by children 6-59 months (%)** | | | | |
| Eggs | 56.3 | 62.2 | 50.2 | 2.11 (1.06, 4.20) .034* |
| Red meat (incl. from hunting) | 56.2 | 52.1 | 60.4 | 0.67 (0.45, 1.02) .063 |
| Fish (incl. river foods) | 45.8 | 54.6 | 36.6 | 2.56 (1.55, 4.23) <.001*** |
| Poultry | 40.1 | 43.8 | 36.3 | 1.53 (0.87, 2.67 ) .138 |
| Dairy products^4^ | 38.0 | 47.3 | 28.4 | 2.95 (1.67, 5.20) <.001*** |

Note. SD = standard deviation.

The table presents outcomes for samples across 4 time points, totaling 1,236 dietary recalls: 618 for mothers, 381 for children aged 24-59 months, and 237 for children 6-23 months old. Frequencies and means are across seasons, to be interpreted as incidences of dietary recalls. Modelling based on mixed effects GLMM with a random intercept (household) accounting for repeated sampling. Accordingly, odds ratios and coefficient estimates account for multiple observations over time, to be interpreted at the participant level.

^1^ ASF are classified in 5-types listed in the bottom section of the table.

^2^ OR = odds ratio, unless β = coefficient if specified. Mid-altitude zone is the reference value. CI = confidence interval.

^3^ *P*-values are a test of association between differences in weekly consumption of ASF and livelihood zone, from logistical mixed-effect models for binary outcomes (OR) and generalized linear mixed models for continuous variables (β).

^4^ Dairy products might include sugary items, as these could not be disaggregated.

Table S3 – Seasonal food groups and animal-source foods consumption and dietary diversity scores of women and children 6-59 months old by north-south aspect in rural Timor-Leste, samples across 4 time points 2017- 2018

| **Seasonal dietary diversity indicators** | **Northern *suku*** | | | | | **Southern *suku*** | | | | |
| --- | --- | --- | --- | --- | --- | --- | --- | --- | --- | --- |
|  | **Dry** | **Wet** | **Early Dry**^1^ | **Dry** | **P-value**^2^ | **Dry** | **Wet**^3^ | **Late**  **wet**^1^ | **Dry** | **P-value**^2^ |
|  | Sep-17 | Jan-18 | Jun-18 | Sep-18 |  | Sep-17 | Jan-18 | Jun-18 | Sep-18 |  |
| Women (n) | 91 | 87 | 86 | 83 |  | 76 | 49 | 72 | 74 |  |
| **DDS-W Food Groups (%)** |  |  |  |  |  |  |  |  |  |  |
| Grains, roots, tuber, plantain | 100 | 100 | 100 | 100 | - | 100 | 100 | 100 | 99 | - |
| Pulses (beans, peas, lentils) | 3 | 8 | 7 | 2 | .296 | 15 | 8 | 8 | 10 | .552 |
| Nuts and seeds | 1 | 5 | 0 | 0 | .194 | 11 | 2 | 0 | 3 | .091 |
| Dairy | 2 | 6 | 1 | 0 | - | 4 | 0 | 0 | 0 | - |
| Meat, poultry, and fish | 36 | 24 | 26 | 37 | .067 | 46 | 43 | 21 | 49 | .002** |
| *Organs* | 1 | 0 | 0 | 1 | .948 | 0 | 6 | 3 | 3 | .589 |
| *Red meat* | 21 | 7 | 12 | 25 | .004** | 32 | 16 | 15 | 29 | .041* |
| *Poultry* | 6 | 4 | 5 | 4 | .915 | 1 | 8 | 6 | 4 | .364 |
| *Fish* | 15 | 15 | 11 | 11 | .597 | 15 | 29 | 4 | 20 | .007** |
| Eggs | 4 | 1 | 5 | 2 | .549 | 7 | 8 | 7 | 4 | .804 |
| Dark green leafy vegetables | 65 | 85 | 74 | 55 | <.001*** | 84 | 83 | 81 | 65 | .026* |
| Other Vit-A rich fruits & veg | 23 | 9 | 19 | 5 | .004** | 24 | 8 | 25 | 15 | .075 |
| Other vegetables | 35 | 29 | 33 | 29 | .745 | 38 | 43 | 33 | 37 | .896 |
| Other fruits | 11 | 10 | 7 | 4 | .271 | 16 | 10 | 11 | 4 | .162 |
| **DDS-W mean (SD)** | 2.8 (1.3) | 2.8 (1.0) | 2.7 (1.0) | 2.3 (0.9) | .011* | 3.4 (1.2) | 3.0 (0.9) | 2.9 (0.9) | 2.8 (1.0) | <.001*** |
| **Low diversity (≤2 FG) (%)** | 51.6 | 46.0 | 59.3 | 57.8 | .203 | 22.4 | 28.6 | 43.1 | 43.2 | .014* |
| Children 6-59 months (n) | 91 | 87 | 86 | 83 |  | 76 | 49 | 72 | 74 |  |
| **DDS Food Groups (%)** |  |  |  |  |  |  |  |  |  |  |
| Grains, roots, and tubers | 100 | 100 | 100 | 100 | - | 96 | 100 | 100 | 99 | - |
| Legumes and nuts | 6 | 6 | 9 | 2 | .311 | 16 | 8 | 4 | 5 | .052 |
| Dairy products | 21 | 14 | 16 | 1 | .010* | 21 | 14 | 8 | 4 | .013* |
| Flesh foods | 34 | 29 | 21 | 34 | .129 | 42 | 35 | 21 | 50 | .002** |
| *Organ meat* | 1 | 1 | 0 | 0 | .975 | 0 | 4 | 1 | 3 | .671 |
| *Red meat* | 21 | 8 | 13 | 19 | .059 | 29 | 14 | 13 | 30 | .022* |
| *Poultry* | 2 | 8 | 4 | 4 | .283 | 1 | 6 | 7 | 7 | .453 |
| *Fish* | 14 | 15 | 7 | 11 | .274 | 13 | 20 | 6 | 18 | .097 |
| Eggs | 10 | 12 | 6 | 4 | .148 | 20 | 27 | 11 | 10 | .033* |
| Vit-A rich fruits & vegetables | 55 | 66 | 70 | 57 | .067 | 83 | 74 | 81 | 70 | .186 |
| Other fruits and vegetables | 20 | 25 | 24 | 30 | .427 | 38 | 37 | 35 | 30 | .629 |
| **DDS mean (SD)** | 2.5 (1.2) | 2.5 (1.1) | 2.5 (0.9) | 2.3 (0.9) | .487 | 3.2 (1.3) | 2.9 (1.0) | 2.6 (0.9) | 2.7 (1.0) | <.001*** |
| **Low diversity (≤2 FG) (%)** | 53.9 | 54.0 | 59.3 | 60.2 | .732 | 34.2 | 32.7 | 48.6 | 47.3 | .106 |

Note. DDS-W = dietary diversity score –women of reproductive age. SD = standard deviation. FG = food groups. DDS = dietary diversity score –infant and young child feeding. ASF are highlighted in grey, with flesh foods disaggregated in four categories in italics.

^1^ The transition season is shown as actual season in each side of the ridgeline.

^2^ P-value is for Walt test performed after i) mixed-effects logistic model of association between the percentage of participants consuming a food group and season by *suku* aspect; and ii) mixed-effects linear model of association between mean DDS-W or DDS and season by *suku* aspect.

^3^ In January 2018, 25 remote households were not interviewed due to accessibility limitations during the heavy rain period.

Figure S5 – Mean (standard error) food groups consumed yesterday among children by age group, comprising 618 dietary recalls across the seasons in both livelihood zones, samples across 4 time points 2017-2018

Note. Sample: Dry (n = 167), Wet (n = 136), Transition (n = 158), Dry (n = 157). Due to the longitudinal nature of the study, in the last iteration there were no children under 18-months, with 19.1% aged 6-23 months and 80.9% 24-59 months old.

Table S4 - Bivariate analyses with each pre-selected covariates for the three outcome variables of child dietary quality, i.e. DDS, MDD and ASF consumed yesterday, samples across 4 time points 2017-2018

| **Predictor variables** | **Child dietary quality outcomes** | | | | |
| --- | --- | --- | --- | --- | --- |
|  | **DDS^1^** | | **MDD^2^** | | **ASF consumed yesterday^2^** |
| Mother-child dyads (n=167) | Coefficient | | Odds Ratio | | Odds Ratio |
| **Mother dietary indicators:** | | | | | |
| DDS-W | 0.55 *** | | 4.07 *** | | 2.43 *** |
| MDD-W No | Reference | |  | |  |
| Yes | 1.11 *** | | 17.81 *** | | 5.43 *** |
| ASF yesterday No | Reference | |  | |  |
| Yes | 0.82 *** | | 6.48 *** | | 29.63 *** |
| **Household characteristics:** | | | | | |
| **Child:** | | | | | |
| **Age group** 6-23 months | Reference | |  | |  |
| 24-59 months | 0.07 | | 0.94 | | 1.19 |
| **Sex**  Female | Reference | |  | |  |
| Male | 0.05 | | 1.19 | | 1.16 |
| **Sick last 2 weeks** No | Reference | |  | |  |
| Yes | 0.15 | | 1.66 | | 1.00 |
| **Diarrhoea last 2w**  No | Reference | |  | |  |
| Yes | 0.02 | | 1.12 | | 0.95 |
| **DDS-IYCF**^3^ |  | |  | | 11.34 *** |
| **MDD-IYCF** No | Reference | |  | |  |
| Yes | 2.05*** | |  | | 25.16 *** |
| **ASF yesterday** No | Reference | |  | |  |
| Yes | 1.25 *** | | 25.58 *** | |  |
| **Mother:** | | | | | |
| **Age group** <30 years | Reference | |  | |  |
| ≥30 years | 0.02 | | 0.94 | | 0.86 |
| **Education** No schooling | Reference | |  | |  |
| Primary | 0.36 * | | 2.85 * | | 1.06 |
| Secondary or + | 0.57 *** | | 5.85 *** | | 2.28 ** |
| **Number of children** 1-4 | Reference | |  | |  |
| >4 | -0.2 | | 0.49 | | 0.61 |
| **Household:** | | | | | |
| **Number members^4^** 3-7 | Reference | |  | |  |
| >7 | -0.30 *** | | 0.60 | | 0.52 ** |
| **Sanitation** Unimproved | Reference | |  | |  |
| Improved | 0.51 *** | | 3.07 ** | | 2.00 * |
| **FIES^5^** Moderate & Severe | Reference | |  | |  |
| Food Secure | 0.34 * | | 2.13 * | | 2.14 * |
| **Wealth Index** Lowest | Reference | |  | |  |
| Middle | 0.05 | | 1.18 | | 1.10 |
| Highest | 0.38 ** | | 2.17 * | | 1.61 |
| **Agro-ecological characteristics:** | |  | |  | |
| **Grows rice** No | Reference | |  | |  |
| Yes | -0.03 | | 1.41 | | 1.21 |
| **Grows maize** No | Reference | |  | |  |
| Yes | 0.03 | | 1.05 | | 0.95 |
| **Grows Vit-A F & Veg^4^** <5 | Reference | |  | |  |
| ≥5 | 0.02 | | 0.94 | | 1.29 |
| **Owns buffalo^4^** <1 | Reference | |  | |  |
| ≥1 | -0.01 | | 1.04 | | 0.87 |
| **Owns chickens^4^** <10 | Reference | |  | |  |
| ≥10 | -0.08 | | 0.77 | | 0.78 |
| **Livelihood zone** Mid-alt. | Reference | |  | |  |
| Coastal | 0.28 * | | 1.51 | | 1.90 ** |
| **Aspect**  North | Reference | |  | |  |
| South | 0.39 ** | | 2.28 * | | 1.81 * |
| **Season**  Dry | Reference | |  | |  |
| Wet | -0.14 | | 0.36 ** | | 0.78 |
| Transition | -0.24 * | | 0.27 *** | | 0.44** |
| Dry | -0.30 ** | | 0.21 *** | | 0.64 |

Note. DDS = dietary diversity score –infant and young children feeding. MDD = minimum dietary diversity –infant and young children feeding. DDS-W = dietary diversity score –women of reproductive age. MDD-W = minimum dietary diversity –women of reproductive age.

The table analyses the sample across four time points, totalling 618 dietary recalls for children 6-59 months old and 618 dietary recalls for mothers. Results from generalized linear mixed models (GLMM)^1^ and mixed-effects logistic model^2^ accounting for households and sampling rounds. Data show Coefficient /Odds Ratio and level of significance (*P* <.05 = *, *P* <.01 = **, *P* <.001 = ***).

^3^ DDS and MDD are correlated and not tested together due to collinearity issues.

^4^ Median set as the cut-off.

^5^ Food insecurity is not included in the multivariate model due to the longitudinal nature of the data vs a single time point collection of an indicator that would certainly vary seasonally.
